# Exosome-mediated transfer of miR-93-5p from cancer-associated fibroblasts confer radioresistance in colorectal cancer cells by downregulating FOXA1 and upregulating TGFB3

**DOI:** 10.1186/s13046-019-1507-2

**Published:** 2020-04-15

**Authors:** Xijuan Chen, Junqi Liu, Qinglan Zhang, Baoxing Liu, Yan Cheng, Yonglei Zhang, Yanan Sun, Hong Ge, Yingqiang Liu

**Affiliations:** 1 Department of Radiation Oncology, the Affiliated Tumor Hospital of Zhengzhou University, No. 127, Dongming Road, Jinshui District, Zhengzhou, 450008 Henan Province People’s Republic of China; 2 Department of Radiation Oncology, the First Affiliated Hospital of Zhengzhou University, Zhengzhou, 450052 People’s Republic of China; 3 Department of Hematology, the Affiliated Tumor Hospital of Zhengzhou University, Zhengzhou, 450008 People’s Republic of China; 4 Department of Chest Surgery, the Affiliated Tumor Hospital of Zhengzhou University, Zhengzhou, 450008 People’s Republic of China; 5 Department of Gynecology, the First Affiliated Hospital of Zhengzhou University, Zhengzhou, 450052 People’s Republic of China; 6 Department of General Surgery, the Affiliated Tumor Hospital of Zhengzhou University, No. 127, Dongming Road, Jinshui District, Zhengzhou, 450008 Henan Province People’s Republic of China

**Keywords:** Colorectal cancer, Cancer-associated fibroblasts, microRNA-93-5p, FOXA1, TGFB3, Radioresistance

## Abstract

**Background:**

Cancer-associated fibroblasts (CAFs) have been intensively studied in recent studies with aims of finding more concrete evidence on their mechanism of involvement in tumor progression, which is currently unknown. CAFs can secrete exosomes which are loaded with proteins, lipids and RNAs, all of which affect tumor microenvironment. The present study identified microRNA-93-5p (miR-93-5p) as a novel exosomal cargo responsible for the pro-tumorigenic effects of CAFs on colorectal cancer (CRC).

**Methods:**

CAFs and normal fibroblasts (NFs) were isolated from cancerous tissues and matched with paracancerous tissues that had been surgically resected from CRC patients. The interaction among miR-93-5p, forkhead box A1 (FOXA1) and TGFB3 was identified through ChIP and dual luciferase reporter assays. The proliferation and apoptosis of SW480 cells co-cultured with CAFs-derived exosomes under irradiation were evaluated by CCK-8, colony formation, and flow cytometric assays. Tumorigenesis of SW480 cells in nude mice was assessed under the irradiation.

**Results:**

FOXA1 was found to be associated with reduced radioresistance in CRC cells and was verified as a target of miR-93-5p. CAFs-derived exosomes contained higher miR-93-5p than those from NFs, which augmented SW480 cell proliferation and rescued them from radiation-induced apoptosis. miR-93-5p was identified as a mediator of the exosomal effects of CAFs on SW480 cells, possibly through downregulating FOXA1 and upregulating TGFB3. FOXA1 could bind to the promoter of TGFB3, thereby inhibiting nuclear accumulation of TGFB3. Also, CAFs-derived exosomes containing miR-93-5p increased the tumor growth of SW480 cells in irradiated nude mice.

**Conclusion:**

The present study identifies miR-93-5p as a specific exosomal cargo that rescues CRC cells against radiation-induced apoptosis.

## Background

Colorectal cancer (CRC) is the third most common cancer worldwide, with 1–2 million new cases diagnosed annually and the fourth leading cause of cancer-related death, with 700,000 deaths reported every year [1]. By 2030, the burden of CRC is speculated to rise by 60% to include 2.2 million new cases and 1.1 million CRC-related deaths [2]. In recent years, radiotherapy is known as a standard preoperative treatment approach to reduce local recurrence, exhibiting promoted apoptosis in response to radiotherapy [3]. However, CRC cells often develop the resistance to radiotherapy, which remains an intractable problem in therapeutic effect and represents a major obstacle to reduce the death of CRC cells [4]. It is reported that cancer-associated fibroblasts (CAFs), recruited from local tissue-resident fibroblasts or pericryptal fibroblasts and distant fibroblast precursors, is involved in therapeutic resistance in CRC cells [5]. Currently, CAFs serve as a target in the anti-cancer therapy due to its contribution to tumorigenesis and malignant behavior [6]. Therefore, our research interests arouse considering the possible mechanism of CAFs in CRC.

It is interesting to note that CAFs could secret exosomes to CRC cells, thus facilitating the progression and metastasis of CRC [7]. Exosomes are attractive targets for cancer treatments due to their small sizes (40 ~ 100 nm) and great impacts on cells [8]. Accumulating evidences have reported that stromal cells-secreted exosomes in the tumor microenvironment play a vital role in cancer progression through the transfer of their cargo, encompassing proteins, and messenger RNAs (mRNAs), and microRNAs (miRNAs), to cancer cells [9, 10]. CAFs-derived exosomes (CAFs-exo) are transferred to CRC cells with elevation in miRNA levels, contributing to proliferation and chemoresistance of CRC cells [11]. miRNAs refer to small non-coding RNA molecules, which act as a regulator in cell proliferation, apoptosis and tumor growth [12]. miRNAs are also implicated in some critical biological processes, including radioresistance [13]. Some miRNAs, such as miR-31, exert great effects on CRC cells resistant to radiotherapy in CAFs by regulating CRC cell proliferation and apoptosis [14]. miR-93 is demonstrated to have impacts on cell proliferation and tumor progression in breast cancer [15], while its role in radioresistance of CRC cells has not been reported. Moreover, Forkhead box protein A1 (FOXA1), a founding member of FOX family of transcription factors, is also proved to participate in the CRC progression [16, 17]. FOXA1 can bind to the promoters of more than 100 genes to influence signaling pathways and cell cycle in human cancers [18], while its specific mechanism in CRC cells resistant to radiotherapy remains largely unknown. Based on the literature and findings, we proposed the hypothesis that CAFs-exo may transfer miR-93-5p to CRC cells. As FOXA1 was predicted to be a target of miR-93-5p by online prediction analyses, we speculated that miR-93-5p could mediate radioresistance in CRC cells by targeting FOXA1. Hence, the current study aims to validate if the aforementioned hypothesis was valid and to further explore the mechanisms by which exosomal miR-93-5p affects the radioresistance in CRC cells through regulation of FOXA1 expression.

## Materials and methods

### Ethics statement

The study was approved by the Ethics Committee of the Affiliated Tumor Hospital of Zhengzhou University and the written informed consent was obtained from all patients. All animal experiments were in line with the Guide for the Care and Use of Laboratory Animal by the National Institutes of Health.

### Study subjects

CRC tissue samples were collected from 75 patients (46 males and 29 females; aged 55–76 years with a mean age of 63.16 ± 5.98 years) who received surgical resection in the Affiliated Tumor Hospital of Zhengzhou University from August 2016 to October 2018. During the operation, 75 pairs of tumor tissues and adjacent normal tissues were harvested and immediately washed with phosphate buffer saline (PBS) containing 20% antibiotics. The tissues were then digested with type I collagenase (Sigma-Aldrich Chemical Company, St Louis, MO, USA) and hyaluronidase (Sigma-Aldrich Chemical Company, St Louis, MO, USA) to isolate NFs and CAFs [7].

### Cell culture

Human normal intestinal epithelial cells (HIEC) and human CRC cells lines, HT-29, SW480, and LoVo were purchased from American Type Culture Collection (Manassas, VA, USA). All cell lines underwent incubation in the Roswell Park Memorial Institute (RPMI) 1640 medium (HyClone Company, Logan, UT, USA) supplemented with 10% fetal bovine serum (FBS; Life Technologies Corporation, Gaithersburg, MD, USA) and 0.2% penicillin and streptomycin. CAFs and NFs were cultured in Dulbecco’s modified Eagle’s medium (DMEM)/F12 medium containing 10% FBS. Cells were then cultured in a 5% CO_2_ incubator (thromo3111, Jinan Beisheng Medical Devices Co., Ltd., Jinan, Shandong, China) at 37 °C.

### Immunofluorescence staining

CAFs and NFs cells were seeded into 6-well plates coated with polylysine, followed by fixation in 4% polyformaldehyde at room temperature for 30 min and incubation with blocking buffer (Beyotime Institute of Biotechnology, Shanghai, China) at 37 °C for 60 min. The samples were incubated with specific primary antibody, rabbit antibodies to α-SMA (ab32575, 1: 200), and FAP (ab53066, 1: 50), FSP1 (ab124805, 1: 500) at 4 °C overnight. All of the above antibodies were purchased from Abcam Inc. (Cambridge, UK). Subsequently, the cells were cultured with fluorescent secondary antibodies, donkey anti-rabbit antibody to Alexa Fluor 594 (A21202, 1: 400) or donkey anti-mouse antibody to Alexa Fluor 488 (A21207, 1: 400), which were provided by the Life Technologies Corporation (Gaithersburg, MD, USA). After incubation avoiding light exposure for 1 h, cells were stained with 4′, 6-diamidino-2-phenylindole (Beyotime Institute of Biotechnology, Shanghai, China) at room temperature for 5 min and photographed under a High Content Screening Imaging System (ImageXpress Micro 4, Molecular Devices, San Jose, CA, USA). Nuclear translocation of TGFB3 was detected using the same procedure mentioned above. The antibodies used were rabbit antibody to TGFB3 (ab15537, 5 μg/ml) and donkey anti-rabbit antibody to Alexa Fluor 594 (A21202, 1: 400).

### Isolation of exosomes

CAFs and NFs cells were cultured in 6-well plates, respectively. Cells in each well were then cultured with 2 mL serum-free DMEM/F12 medium for 2 h, when the confluence reached 80–90%. The exosomes were harvested from CAFs-culture medium (CM) or NFs-CM by filtration through a 0.22 μm filter, followed by ultracentrifugation at 100000×g for 90 min. The concentrated material underwent centrifugation at 100000×g (4 °C) for 60 min. The resulting pellet was re-suspended and pelleted again. The final pellet was re-suspended in a small volume of PBS. Exosomes were stored at − 20 °C until further use.

### Transmission electron microscope (TEM)

After the ultracentrifugation of exosomes, the precipitation was fixed with the mixture of 2% polyformaldehyde and 2.5% glutaraldehyde at 4 °C for 1 h, and with 1% osmic acid for 1.5 h. Following dehydration with gradient alcohol, immersion in epoxy resin overnight and embedding, the samples were polymerized at 35 °C, 45 °C, and 60 °C for 24 h and sectioned. The sections were stained with lead-uranium and observed under a TEM (H-600, Hitachi, Tokyo, Japan).

### Nanoparticle tracking analysis (NAT)

Size distributions and quantification of exosomes were determined by measuring the rate of Brownian motion using a Nanoparticle tracer analyzer (Malvern, Malvern, UK). The diluted samples at concentration of (1–9) × 10^8^ cells/ml were detected by the machine. The appropriate background gray level was selected by the operation software. The particle trajectory was recorded and the concentration and particle size distribution of the diluted samples were output. The concentration of exosomes in the original solution was calculated by dilution ratio.

### Western blot analysis

The total protein was extracted using radio immunoprecipitation assay (R0010, Beijing Solarbio Science & Technology Co., Ltd., Beijing, China) containing phenylmethylsulfonyl fluoride. Total proteins were subjected to 10% sodium dodecyl sulfate-polyacrylamide gel electrophoresis, and transferred onto the polyvinylidene fluoride membrane (Millipore, Billerica, MA, USA). The membrane underwent incubation with the primary antibodies, rabbit antibodies to CD63 (ab118307, 1: 50), CD81 (ab109201, 1: 1000), TSG101 (ab125011, 1: 1000), GRP94 (ab13509, 1: 1000), FOXA1 (ab151522, 1: 500), TGFB3 (ab227711, 1: 5000), TGF-β1 (ab92486, 2 μg/mL), Smad3 (ab40854, 1: 1000), p-Smad3 (ab63403, 1: 2000) and GAPDH (ab8245, 1: 10000) at 4 °C overnight. Subsequently, the membrane was supplemented with horseradish peroxidase-labeled Immunoglobulin G (IgG; ab205719, 1: 2000) as the secondary antibody for incubation for 1 h and visualized using an enhanced chemiluminescence kit (BB-3501, Amersham Pharmacia Biotech, Chicago, IL, USA). All of the aforementioned antibodies were purchased from Abcam Inc. (Cambridge, MA, USA). Afterwards, the samples were photographed using the IS gel image analysis system and analyzed using the Image J.

### Co-culture of CRC cells and exosomes

The exosomes were labeled with PKH67 (Sigma-Aldrich Chemical Company, St Louis, MO, USA) to monitor the interaction between CAFs-exo and NFs-derived exosomes (NFs-exo) with SW480 cells. After co-culture with PKH67-labeled CAFs-exo and NFs-exo for 24 h in a 5% CO_2_ incubator at 37 °C, SW480 cells were observed with the use of a Nikon Eclipse Ti confocal laser scanning microscope.

### Cell treatment

All plasmids were purchased from Guangzhou RiboBio Co., Ltd. (Guangzhou, Guangdong, China). SW480 cells were suspended in serum-free RPMI 1640 medium and seeded in 6-well plate. The transfection was performed using Lipofectamine 2000 (Invitrogen, Carlsbad, CA, USA), according to the manufacturer’s instructions. Following a 24 h transfection, cells were used for the follow-up experiments. Mimic-NC and miR-93-5p mimic plasmids were transfected into CAFs by the same method. After transfection for 24 h, the exosomes were isolated from CAFs. Next, exosomes were co-cultured with SW480 cells or transfected SW480 cells for the subsequent experiments.

### Irradiation in vitro

Cells in each group were cultured in disposable T25 culture flasks (5 × 10^6^ cells/flask) in a 5% CO_2_ incubator at 37 °C for 16 h. Prior to irradiation, the culture flasks were filled with culture medium, and the condensate plate was used as medium to set up a built-up area (1.5 cm). Cells were irradiated with medical electron linear accelerator. The total dose was 6 Gy and the dose rate was 5 Gy/min. Source-axis distance was 100 cm, and culture continued for 48 h following irradiation.

### RNA isolation and quantitation

Total RNA was extracted from cells and tissues using a RNeasy Mini Kit (Qiagen, Valencia, CA, USA). Next, total RNA of mRNA and lncRNA was reversely transcribed into cDNA using a reverse transcription kit (RR047A, Takara Bio Inc., Otsu, Shiga, Japan), and the total RNA of miRNA was reversely transcribed into a cDNA using miRNA First Strand cDNA Synthesis (Tailing Reaction) kit (Shanghai Sangon Biotechnology Co., Ltd., Shanghai, China). According to the instructions provided on the SYBR® Premix Ex Taq™ II (Perfect Real Time) kit (DRR081, Takara Bio Inc., Otsu, Shiga, Japan), reverse transcription quantitative polymerase chain reaction (RT-qPCR) was conducted for mRNA and lncRNA using a real-time PCR instrument (ABI 7500, ABI, Foster City, CA, USA). The general negative primers of miRNAs and the upstream primers of internal reference U6 were provided in the miRNA First Strand cDNA Synthesis (Tailing Reaction) kit. Other primers were synthesized by Shanghai Sangon Biotechnology Co., Ltd. (Shanghai, China) (Table 1). The glyceraldehyde-3-phosphate dehydrogenase (GAPDH) and U6 were considered as the internal references. The expression ratio of target gene between the experimental and control groups was calculated using the 2^-ΔΔCt^ method.

**Table 1:** Primer sequences for RT-qPCR

RNA	Forward (5′-3′)	Reverse (5′-3′)
miR-93-5p	GCAGCAAACTTCTGAGACAC	GTGCAGGGTCCGAGGTATTC
FOXA1	GCAATACTCGCCTTACGGGCT	TACACACCTTGGTAGTACGCC
TGFB3	ATGCCAAAGAAATCCATAAATTC	GAAGCGGAAAACCTTGGAGGTA
TGF-β1	CACCATCCATGACATGAACC	TCATGTTGGACAACTGCTCC
Smad3	ACGCAGAACGTGAACACCAA	GCTGTGAAGCGTGGAATGTC
GAPDH	ACAACCTTTGGTATCGTGGAAGG	GCCATCACGCCACAGTTTC

### Cell counting kit-8 (CCK-8)

After receiving their corresponding treatments in the study, SW480 cells were harvested and seeded into 96-well plates (1 × 10^5^ cells/mL, 100 μL) for a 24-h incubation with 5% CO_2_ at 37 °C. To test the cell viability, each well was added with 10 μL CCK-8 reagent at 24 h, 48 h, and 72 h and incubation was carried out. After 4 h, the optical density (OD) value was measured at the wavelength of 450 nm using a microplate reader.

### Colony formation assay

For soft agar colony formation assay, 2000 cells were seeded in 0.3% agar on a base of 2 mL 0.6% agar (Gibco, Carlsbad, CA, USA) in a 6-well plate. Culture dishes were transferred sequentially to a refrigerator at 4 °C for 10 min, and then to the cell culture incubator at 37 °C for 14 d. The colonies (more than 50 cells) were inspected and photographed under a microscope. Three parallel wells were set in the experiment, with the mean value obtained.

### Flow cytometry

Single-cell suspensions were fixed in 70% precooling ethanol overnight at 4 °C, washed twice with PBS, and incubated with 1 mL propidium (PI, 50 mg/L)/RNAase (Sigma-Aldrich Chemical Company, St Louis, MO, USA) for 30 min under dark conditions. Cells were then evaluated using a flow cytometer (Gallios, Beckman Coulter, Shanghai, China) at 488 nm. To analyze apoptosis rates, the cell suspension was incubated with 10 μL Annexin V-fluorescein isothiocyanate (FITC) and 5 μL PI without light exposure for 15 min and were analyzed immediately with the use of a flow cytometry.

### Dual luciferase reporter gene assay

Luciferase reporter vectors were constructed by inserting the three-prime untranslated regions (3’UTR) of FOXA1 downstream of the luciferase gene in a pGL3-control (Beijing Huayueyang Biotechnology Co., Ltd., Beijing, China). A site-specific mutation at the miR-93-5p binding site was created to make a target mutant form (mut) based on the FOXA1-wild type (wt). The correctly sequenced luciferase reporter plasmids of wt and mut were co-transfected with miR-93-5p mimic into the HEK-293 T cells.

The TGFB3 promoter luciferase reporter plasmid (PGL3-basic-TGFB3P) was inserted into the TGFB3 promoter sequence encoding − 45 ~ − 39, with PGL3-basic used as carrier, 5′-GACGTCA-3, which was constructed by Shanghai Generay Biotech Co., Ltd. (Shanghai, China). The PGL3-basic-TGFB3P plasmids were co-transfected with oe-NC and oe-FOXA1 plasmids into CRC cells, respectively. After transfection for 48 h, cells were lysed and incubated at 25 °C for 20 min.

A dual Luciferase Reporter Assay System kit (Promega Company, Madison, WI, USA) was used to detect the activity of firefly luciferase (M1) and of renilla luciferase (M2) in cells of each group. Luciferase activity of target gene and promoter was expressed as M2/M1.

### Chromatin immunoprecipitation (ChIP) assay

The ChIP kit (Merck Millipore, Billerica, MA, USA) was used to detect the enrichment of FOXA1 in TGFB3 promoter region. According to the instructions, primers were designed based on the promoter sequence of TGFB3: 5′-TGCGCCCCCTCTACATTG-3′ and 5′ -GGTTCGTGGACCCATTTCC-3′, and synthetized by the Invitrogen (Shanghai, China).

### Tumor xenografts in nude mice

Before animal experiments, a total of 1.5 × 10^6^ SW480 cells were cultured in (1) serum-free medium, (2) serum-free medium containing 100 μg agomir-NC-exo or (3) serum-free medium containing 100 μg agomir-93-5p-exo. After 12 h, the SW480 cells were washed with PBS for the removal of excessive exosomes. A total of 30 specific-pathogen-free (SPF) BALB/c nude mice (aged 3–4 weeks) were obtained from the Shanghai SLAC Laboratory Animal Co., Ltd. (Shanghai, China). Nude mice were injected with the SW480 cells with different treatments as mentioned above. After 2 weeks, the tumors were 0.5 cm^3^ in size, and subjected to local X-ray irradiation (once every 2 days, 2 Gy/time, totally 3 times). Tumor volume was recorded every 3 days. After 15 days of irradiation, nude mice were euthanized using barbiturate overdose, and the tumors were isolated for immunohistochemistry.

### Immunohistochemistry

Serial sections (4 μm) were cut from formalin-fixed and paraffin-embedded xenograft tissue samples for Immunohistochemistry in accordance with a streptavidin peroxidase kit (Beijing Zhongshan Biotechnology Co., Ltd., Beijing, China). The sections underwent incubation with the rabbit antibodies to Bax (ab97779, 1: 500), Bcl2 (ab38898, 1: 5000), and goat anti-rabbit antibody to IgG (ab150077, 1: 500). Finally, the samples were observed and photographed under an inverted fluorescence Microscope (IX70, Olympus, Tokyo, Japan).

### Statistical analysis

The data was analyzed using SPSS 21.0 software (IBM Corp., Armonk, NY, USA). The measurement data was expressed as mean ± standard deviation. The comparison of measurement data conforming to normal distribution and homogeneous variance with paired design between two groups was conducted by paired *t*-test. The comparison of measurement data conforming to normal distribution and homogeneous variance with unpaired design between two groups was conducted using the unpaired *t*-test. The comparison among multiple groups was assessed by one-way analysis of variance (ANOVA), followed by Tukey’s post hoc test. The comparisons of data at different time points were performed by the repeated measures ANOVA, followed by Bonferroni’s post hoc test. The relationship between two indicators was analyzed using the Pearson correlation analysis. *p* value < 0.05 was indicative of statistical significance.

## Results

### FOXA1 is downregulated in CRC and inhibits chemoresistance of CRC cells

**Fig. 1:**
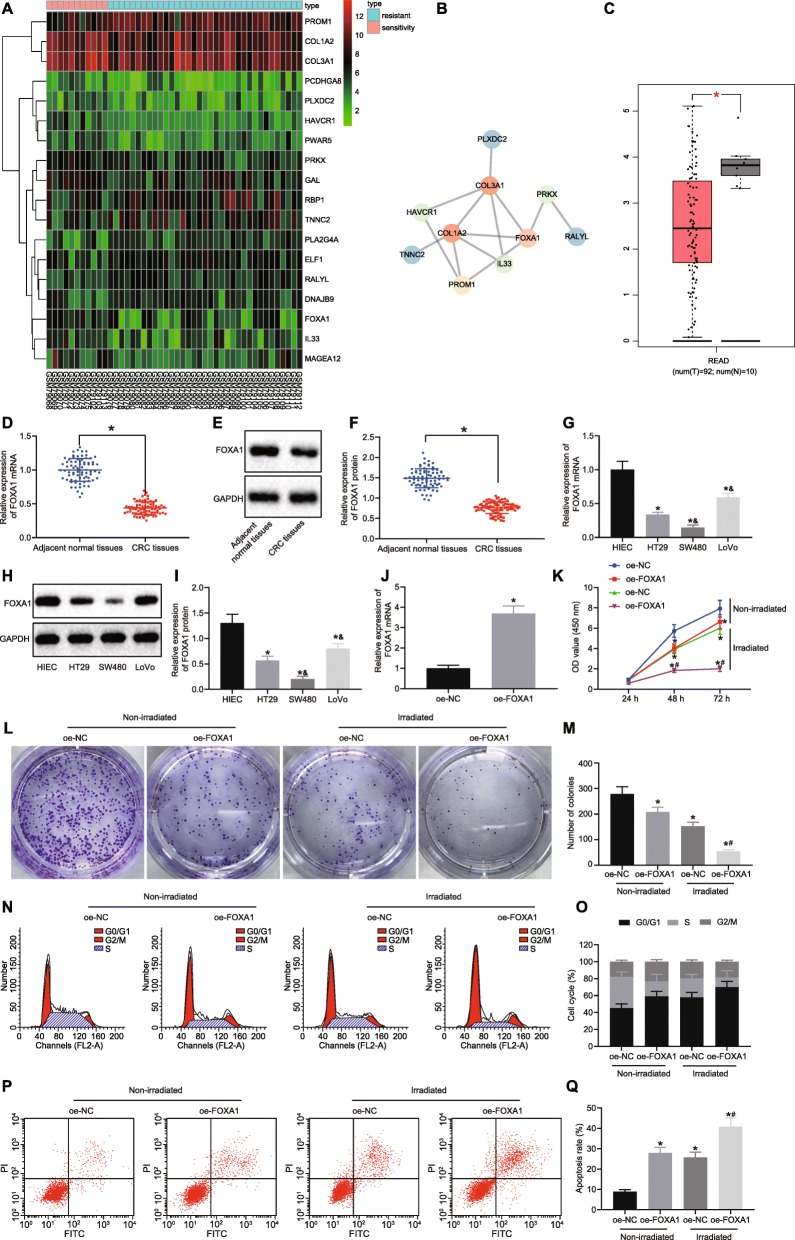
FOXA1 is poorly expressed in CRC tissues and cell lines. **a** Differential expression analysis for CRC-related microarray data GSE3493. The X axis indicated the sample number and the Y axis indicated the DEGs. The upper right histogram indicated color gradation. **b** Difference analysis was carried out using “limma” package of R language with |log FoldChange| > 1 and *p* < 0.05 as screening criteria for DEGs. The STRING database (https://string-db.org/) was used to analyze the intersection and association of DEGs. Each circle in the graph represented one gene, and the lines between circles represented the interaction between genes. The darker the color was, the higher the core level of the gene was in the network. **c** FOXA1 expression analyzed by TCGA database. The X axis indicated the disease type and the Y axis indicated the gene expression. The red referred to the tumor samples, and the gray referred to the normal samples. **d** FOXA1 mRNA expression in CRC tissues (*n* = 75) and adjacent normal tissues (n = 75) determined by RT-qPCR, normalized to GAPDH. **e** FOXA1 protein band pattern in CRC tissues (n = 75) and adjacent normal tissues (n = 75) detected by Western blot analysis. **f** FOXA1 protein expression in CRC tissues (n = 75) and adjacent normal tissues (n = 75) determined by Western blot analysis, normalized to GAPDH. **g** FOXA1 mRNA expression in HIEC and CRC cells determined by RT-qPCR, normalized to GAPDH. **e** FOXA1 protein band pattern in HIEC and CRC cells detected by Western blot analysis. **f** FOXA1 protein expression in HIEC and CRC cells determined by Western blot analysis, normalized to GAPDH. SW480 cells were treated with FOXA1 overexpression plasmid (with oe-NC as control) and exposed to irradiation (with non-irradiated cells as control). **k** Cell viability in SW480 cells detected using CCK-8 assay. **k** Colony formation ability of SW480 cells detected using colony formation assay. **m** The number of colonies of SW480 cells detected using colony formation assay. **n** Cell cycle in SW480 cells detected using flow cytometry. **o** The proportion of SW480 cells detected using flow cytometry. **p** Apoptosis of SW480 cells detected using flow cytometry. **q** Apoptotic rate of SW480 cells detected using flow cytometry. Values obtained from three independent experiments in triplicate are expressed as mean ± SD and analyzed by paired t test between CRC tissues and adjacent normal tissues, by unpaired t test between CRC cells and HIEC cells, by one-way ANOVA followed by Tukey’s post hoc test among multiple groups, and by repeated measures ANOVA followed by Bonferroni at different time points. ^*^p < 0.05 compared with adjacent normal tissues, HIEC cells, or SW480 cells treated with oe-NC plasmids; ^&^*p* < 0.05 compared with HT-29 cell lines; ^#^*p* < 0.05 compared with non-irradiated cells

Differential analysis was conducted for radiosensitive and radio-resistant CRC-related microarray data GSE3493, which identified 18 genes with significant difference in expression in radioresistant samples relative to radiosensitive samples (Fig. 1a). Subsequently, String was used to plot a network map between those genes, indicating that FOXA1, COL3A1 and COL1A2 were at the core of the network map (Fig. 1b). Among these genes, the FOXA1 expression in radioresistant samples presented with the most evident difference (Table 2). Moreover, FOXA1 expression in CRC-related data in TCGA database was analyzed, which revealed that FOXA1 was significantly reduced in CRC samples (Fig. 1c).

**Table 2:** Differential expression of core genes

Symbol	LogFC	AveExpr	t	*P* value
FOXA1	−1.624827725	5.050121575	−2.588786061	0.012687202
COL1A2	−1.136358405	9.49587451	−2.580657536	0.012951912
COL3A1	−1.188253693	10.18747811	−2.369404832	0.021857387

RT-qPCR and Western blot analysis revealed that FOXA1 was poorly expressed in CRC tissues (Fig. 1d–f). FOXA1 expression was lower in CRC cell lines than that in intestinal epithelial cell line HIEC, and was the lowest in the SW480 cell line (Fig. 1g–i). Thus, SW480 cells were selected for the subsequent experiments.

RT-qPCR showed increased FOXA1 expression in SW480 cells transfected with FOXA1 overexpression plasmid (Fig. 1j). The transfected cells were irradiated, with the non-irradiated cells serving as the control. CCK-8 assay and colony formation assay showed that restored FOXA1 diminished cell viability and colony formation in both irradiated and non-irradiated cells (*p* < 0.05). After irradiation, cell viability and colony formation were inhibited in SW480 cells and significantly suppressed in cells with overexpressed FOXA1 (*p* < 0.05; Fig. 1k–m). Flow cytometry showed that upregulation in FOXA1 increased the proportion of cells in G1 phase, decreased the proportion of cells in S phase, and elevated the apoptotic rate. Following irradiation, the changes of these indexes were more significant in cell treated with overexpressed FOXA1 (*p* < 0.05; Fig. 1n–q). The data obtained indicated that FOXA1 expression was decreased in CRC tissues and cells, and elevated FOXA1 resulted in the inhibition of chemoresistance of CRC cells.

### FOXA1 is a target gene of miR-93-5p

**Fig. 2:**
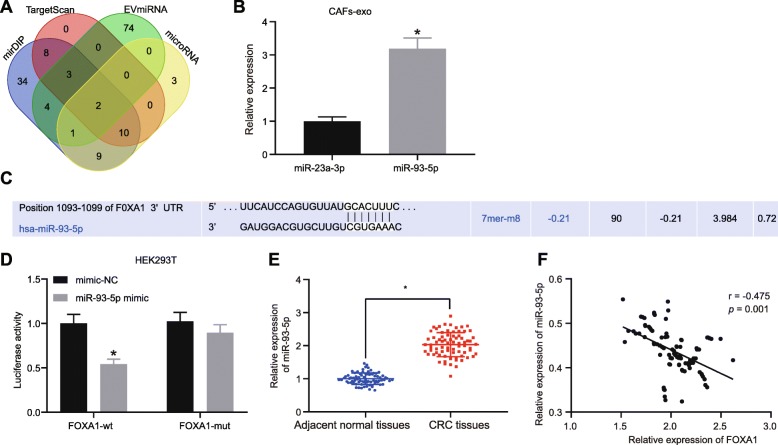
miR-93-5p targets and negatively regulates FOXA1. **a** Predicted results of miRNAs that regulated FOXA1 in TargetScan, EVmiRNA, mirDIP, and microRNA databases. **b** miR-93-5p expression in CAFs-exo determined by RT-qPCR, normalized to GAPDH. **c** Prediction binding sites between miR-93-5p and FOXA1. **d** Luciferase activity of FOXA1-wt and FOXA1-mut in the presence of miR-93-5p mimic or mimic NC detected using dual-luciferase reporter gene assay. **e** miR-93-5p expression in CRC tissues (n = 75) and adjacent normal tissues (n = 75) determined by RT-qPCR, normalized to GAPDH. **f** Correlation between miR-93-5p and FOXA1 analyzed by Pearson. Values obtained from three independent experiments in triplicate are expressed as mean ± SD and analyzed by paired t test between CRC tissues and adjacent normal tissues and by unpaired t test between two groups. ^*^p < 0.05 compared with adjacent normal tissues, or SW480 cells treated with mimic-NC plasmids

The upstream regulation mechanism of FOXA1 was further explored through prediction of miRNAs that may regulate FOXA1 using mirDIP, EVmiRNA, and microRNA databases (Fig. 2a). Based on the findings, there were two miRNAs, miR-93-5p and miR-23a-3p, in the intersection of predicted results. The expression of miRNAs was further measured in the CAFs-exo, which revealed that miR-93-5p expression was higher than miR-23a-3p expression (Fig. 2b). Targetscan, an online analysis website, revealed that there exists specific binding sites between miR-93-5p and FOXA1 (Fig. 2c). Dual-luciferase reporter gene assay verified that FOXA1 was the target gene of miR-93-5p. It was found that luciferase activity of FOXA1-wt instead of FOXA1-Mut was reduced in the presence of miR-93-5p mimic (Fig. 2d). RT-qPCR revealed an elevation in miR-93-5p expression in CRC tissues (*p* < 0.05; Fig. 2e). The correlation analysis showed that miR-93-5p expression was negatively correlated with the FOXA1 expression in CRC tissues (r = − 5.517, *p* < 0.05; Fig. 2f). Overall, these results suggested that miR-93-5p could target FOXA1.

### CAFs-derived exosomes upregulates miR-93-5p and promotes chemoresistance of CRC cells

**Fig. 3:**
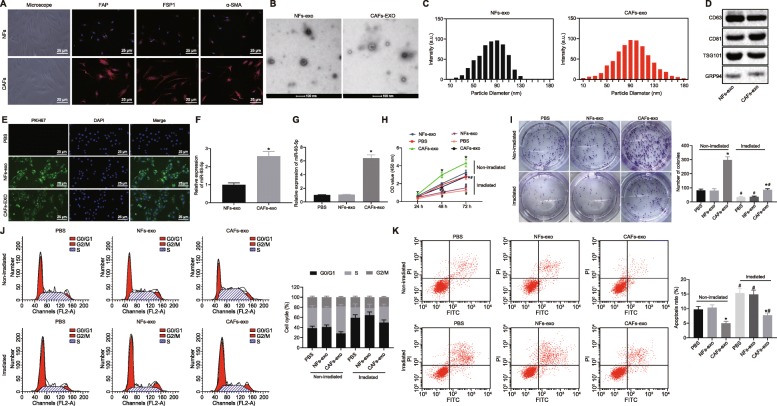
CAFs-derived exosomes enhance chemoresistance of CRC cells by upregulating miR-93-5p. **a** Morphology of isolated fibroblasts observed under an optical microscope and positive expression of α-SMA, FAP and FSP1 detected by immunofluorescence staining (× 400). **b** Ultrastructure of exosomes observed under the TEM (scale bar = 100 nm). **c** NTA analysis of exosome size. **d** Expression of exosome surface marker proteins CD63, CD81 TSG101 and negative marker GRP94 measured by Western blot analysis. **e** The uptake of exosomes by SW480 cells observed under a laser confocal microscope (× 400). **f** miR-93-5p expression in CAFs-exo and NFs-exo determined by RT-qPCR, normalized to GAPDH. **g** miR-93-5p expression in SW480 cells co-cultured with CAFs-exo and NFs-exo determined by RT-qPCR, normalized to GAPDH. **h** Cell viability in SW480 cells detected using CCK-8 assay. **i** Colony formation ability of SW480 cells detected using colony formation assay. **j** Cell cycle distribution in SW480 cells detected using flow cytometry. **k** Apoptosis of SW480 cells detected using flow cytometry. Values obtained from three independent experiments in triplicate are expressed as mean ± SD and analyzed by unpaired t test between two groups, by one-way ANOVA followed by Tukey’s post hoc test among multiple groups, and by repeated measures ANOVA followed by Bonferroni at different time points. ^*^p < 0.05 compared with SW480 cells co-cultured with NFs-exo or CAFs-CM; ^#^*p* < 0.05 compared with non-irradiated cells

CAFs and NFs were slender and fibrous, when observed under the microscope. Immunofluorescence staining showed that the expression of specific marker proteins α-SMA, FAP and FSP1 in CAFs was high in CAFs (Fig. 3a). The exosomes of CAFs (CAFs-exo) and NFs (NFs-exo) were separated. Under the TEM, it was found that the separated vesicles were round or oval membrane vesicles with discoid structure and complete capsule (Fig. 3b). NTA analysis revealed that the separated vesicles had an average particle size of 50–100 nm (Fig. 3c). Western blot analysis revealed that the isolated vesicles expressed exosome surface marker proteins, CD63, CD81, and TSG101, and that they appreciably poorly expressed negative marker GRP94 (Fig. 3d). These findings were suggestive of the successful isolation of exosomes.

The uptake of exosomes by SW480 cells was observed under the laser confocal microscope. No fluorescence signal was detected in the PBS group, while green fluorescence was observed in the cytoplasm of SW480 cells co-cultured with CAFs-exo and NFs-exo; however, there was no significant difference observed in fluorescence intensity (*p* > 0.05; Fig. 3e). RT-qPCR displayed that miR-93-5p expression in CAFs-exo was significantly higher than that in NFs-exo (*p* < 0.05; Fig. 3f). In addition, SW480 cells co-cultured with CAFs-exo presented with higher expression of miR-93-5p in comparison with SW480 cells co-cultured with PBS as revealed by RT-qPCR. However, there was no significant difference observed in miR-93-5p expression in SW480 cells co-cultured with NFs-exo (Fig. 3g).

SW480 cells after co-culture were exposed to irradiation. CCK-8 assay and colony formation assay revealed that following irradiation, cell viability and colony formation reduced (*p* < 0.05). Compared with SW480 cells co-cultured with PBS, cell viability and colony formation presented with no significant difference in cells co-cultured with NFs-exo, but promoted in cells co-cultured with CAFs-exo (*p* < 0.05; Fig. 3h–i).

Flow cytometry showed increased proportion of cells in G1 phase and apoptotic rate, and decreased proportion of cells in S phase following irradiation (*p* < 0.05). Compared with SW480 cells co-cultured with PBS, the proportion of cells in G1 phase and S phase showed no evident difference in cells co-cultured with NFs-exo, while cells co-cultured with CAFs-exo revealed reduced the proportion of cells in G1 phase and apoptotic rate, and elevated proportion of cells in S phase (*p* < 0.05; Fig. 3j–k). These findings suggested that CAFs-exo promoted chemoresistance of CRC cells through the upregulation of miR-93-5p.

### CAFs promoted chemoresistance of CRC cells by delivering miR-93-5p via exosomes

**Fig. 4:**
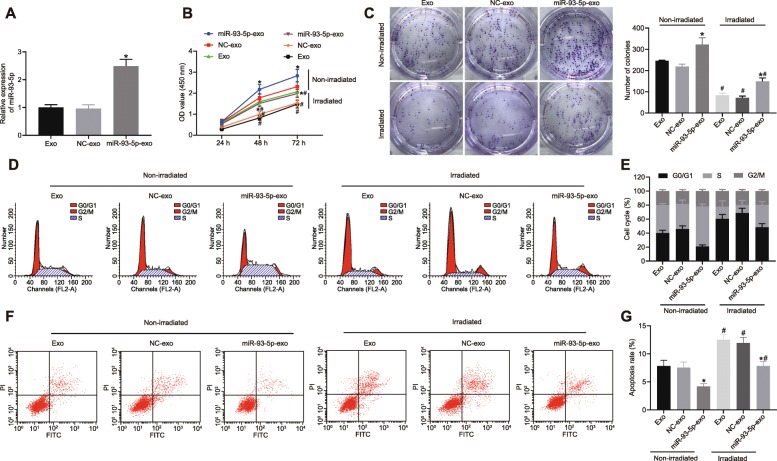
CAFs enhance chemoresistance of CRC cells by delivering miR-93-5p via exosomes. The exosomes were isolated from CAFs overexpressing miR-93-5. Next, SW480 cells were co-cultured with NC-exo and miR-93-5-exo and exposed to irradiation. **a** miR-93-5p expression in CAFs-exo determined by RT-qPCR, normalized to GAPDH. **b** Cell viability in SW480 cells detected using CCK-8 assay. **c**–**d** Colony formation ability of SW480 cells detected using colony formation assay. **e**–**f** Cell cycle distribution in SW480 cells detected using flow cytometry. **g**–**h** Apoptosis of SW480 cells detected using flow cytometry. Values obtained from three independent experiments in triplicate are expressed as mean ± SD and analyzed by one-way ANOVA followed by Tukey’s post hoc test among multiple groups, and by repeated measures ANOVA followed by Bonferroni at different time points. ^*^p < 0.05 compared with SW480 cells co-cultured with NC-exo; ^#^*p* < 0.05 compared with non-irradiated cells

Following transduction of miR-93-5 overexpression in CAFs, the exosomes were isolated. RT-qPCR showed that CAFs with upregulated miR-93-5 were also observed to have elevated miR-93-5p expression in exosomes (*p* < 0.05; Fig. 4a). The SW480 cells were co-cultured with NC-exo and miR-93-5-exo secreted by CAFs and exposed to irradiation. CCK-8 assay and colony formation assay revealed that after irradiation, cell viability and colony formation were reduced (*p* < 0.05). SW480 cells co-cultured with miR-93-5-exo presented the enhanced cell viability and colony formation (*p* < 0.05; Fig. 4b–d). Flow cytometry showed that irradiation induced cell cycle arrest and apoptosis (*p* < 0.05). SW480 cells co-cultured with miR-93-5-exo presented with facilitated G1/S cell cycle transition and inhibited apoptosis (*p* < 0.05; Fig. 4e–h). The data revealed that CAFs promoted chemoresistance of CRC cells by delivering exosomal miR-93-5p.

### CAFs carrying exosomal miR-93-5p promotes chemoresistance of CRC cells by binding to FOXA1

**Fig. 5:**
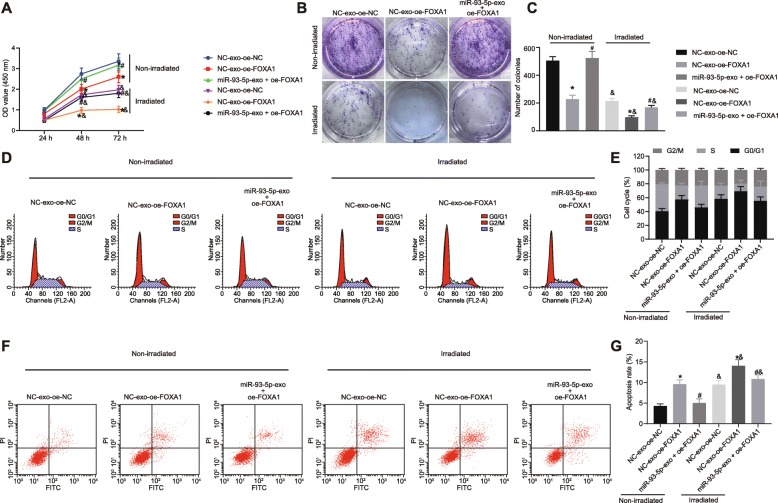
CAFs carrying exosomal miR-93-5p facilitate chemoresistance of CRC cells. SW480 cells were transfected with FOXA1 overexpression plasmid and co-cultured with miR-93-5-exo under exposure to irradiation. **a** Cell viability in SW480 cells detected using CCK-8 assay. **b**–**c** Colony formation ability of SW480 cells detected using colony formation assay. **d**–**e** Cell cycle distribution in SW480 cells detected using flow cytometry. **f**–**g** Apoptosis of SW480 cells detected using flow cytometry. Values obtained from three independent experiments in triplicate are expressed as mean ± SD and analyzed by one-way ANOVA followed by Tukey’s post hoc test among multiple groups, and by repeated measures ANOVA followed by Bonferroni at different time points. ^*^p < 0.05 compared with SW480 cells treated with NC-exo + oe-NC; ^*^p < 0.05 compared with SW480 cells treated with NC-exo + oe-FOXA1; ^&^*p* < 0.05 compared with non-irradiated cells

The exosome overexpressing miR-93-5p were co-cultured with SW480 cells overexpressing FOXA1 and exposed to irradiation. CCK-8 assay and colony formation assay presented that after irradiation, cell viability and colony formation were reduced (*p* < 0.05). SW480 cells transfected with FOXA1 overexpression plasmid and co-cultured with NC-exo showed suppressed cell viability and colony formation, and SW480 cells transfected with FOXA1 overexpression plasmid and co-cultured with miR-93-5p-exo showed the opposite results (*p* < 0.05; Fig. 5a–c). Flow cytometry showed that irradiation caused G1/S cell cycle transition delay and apoptosis promotion (*p* < 0.05). Cells transfected with FOXA1 overexpression plasmid and co-cultured with NC-exo caused G1/S cell cycle transition delay, and SW480 cells transfected with FOXA1 overexpression plasmid and co-cultured with miR-93-5p-exo promoted G1/S cell cycle transition (*p* < 0.05; Fig. 5d–g). Thus, the data implied that CAFs carrying exosomal miR-93-5p facilitated chemoresistance of CRC cells through downregulating FOXA1.

### miR-93-5p activates the TGF-β signaling pathway by inhibiting FOXA1

**Fig. 6:**
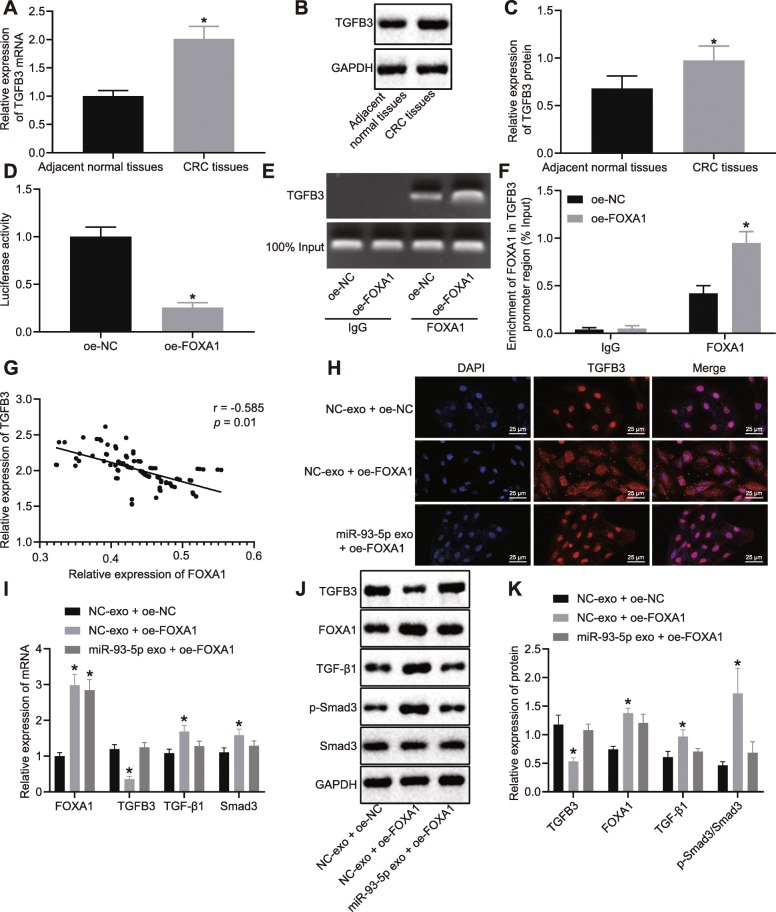
miR-93-5p promotes the activation of TGF-β signaling pathway by inhibiting FOXA1. SW480 cells were transfected with FOXA1 overexpression plasmid and co-cultured with miR-93-5-exo. **a** TGFB3 mRNA expression in CRC tissues (*n* = 75) and adjacent normal tissues (n = 75) determined by RT-qPCR, normalized to GAPDH. **b** TGFB3 protein band pattern in CRC tissues (n = 75) and adjacent normal tissues (n = 75) detected by Western blot analysis. **c** TGFB3 protein expression in CRC tissues (n = 75) and adjacent normal tissues (n = 75) determined by Western blot analysis, normalized to GAPDH. **d** Binding of FOXA1 to TGFB3 promoter detected by dual luciferase reporter gene assay. **e**–**f** Enrichment of FOXA1 in the promoter region of TGFB3 detected by ChIP assay. **g** Analysis of the correlation between FOXA1 and TGFB3 expression in CRC. **h** Detection of nuclear translocation of TGFB3 by immunofluorescence staining (400 ×). Green represented TGFB3 protein, red referred to cells and yellow referred to expression of TGFB3 in the nucleus. **i** mRNA expression of FOXA1, TGFB3, TGF-β1, and Smad3 in SW480 cells determined by RT-qPCR, normalized to GAPDH. **j**–**k** Protein bands and protein levels of FOXA1, TGFB3, TGF-β1, and Smad3 in SW480 cells detected by Western blot analysis, normalized to GAPDH. Values obtained from three independent experiments in triplicate are expressed as mean ± SD and analyzed by one-way ANOVA followed by Tukey’s post hoc test among multiple groups. ^*^p < 0.05 compared with SW480 cells treated with oe-NC or NC-exo + oe-NC

It has been demonstrated that downregulation of FOXA1 could upregulate TGFB3 to modulate TGF-β signaling pathway [19]. RT-qPCR and Western blot analysis revealed that TGFB3 was highly expressed in CRC tissues (*p* < 0.05; Fig. 6a–c). Dual-luciferase reporter gene assay was performed to detect the binding of FOXA1 to TGFB3, which showed that that the luciferase activity of TGFB3 was inhibited after restoration of FOXA1 (*p* < 0.05; Fig. 6d). ChIP assay demonstrated that elevated FOXA1 increased the enrichment of TGFB3 promoter region with FOXA1 (Fig. 6e–f). The correlation analysis showed that FOXA1 was negatively correlated with the TGFB3 in CRC tissues (r = − 3.421, *p* < 0.05; Fig. 6g). Immunofluorescence staining presented that the fluorescence intensity of TGFB3 was increased and mainly distributed in the nucleus in cells treated with oe-NC, while the fluorescence intensity TGFB3 was decreased, mainly distributed in the cytoplasm of cells overexpressing FOXA1. These results suggested that FOXA1 could negatively regulate the transcription of TGFB3 and prevented its translocation into the nucleus.

In addition, RT-qPCR and Western blot analysis displayed that FOXA1 expression was elevated, and expression of TGFB3, TGF-β1, and Smad3 was reduced in cells transfected with FOXA1 overexpression plasmid and co-cultured with NC-exo, while these results were opposite in SW480 cells transfected with FOXA1 overexpression plasmid and co-cultured with miR-93-5p-exo (*p* < 0.05; Fig. 6i–k). The abovementioned findings proved that miR-93-5p activated the TGF-β signaling pathway by inhibiting FOXA1.

### CAFs carrying exosomal miR-93-5p promotes chemoresistance of CRC cells in vivo by regulating FOXA1

**Fig. 7:**
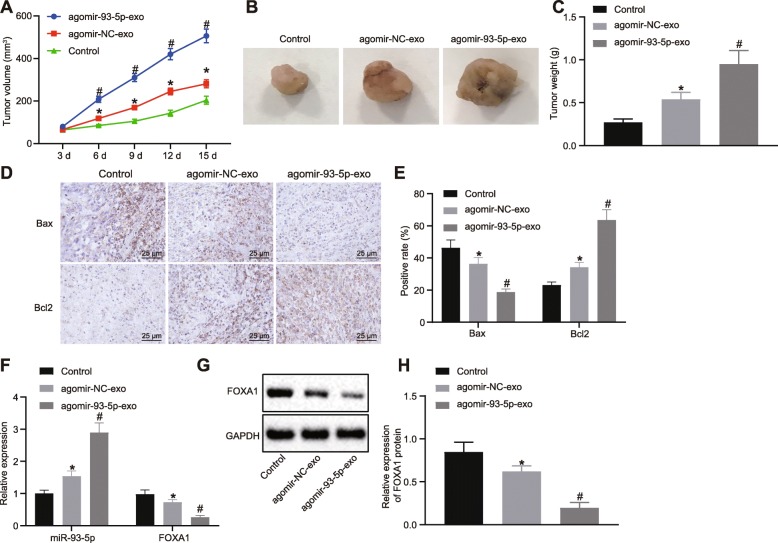
CAFs carrying exosomal miR-93-5p promote chemoresistance of CRC cells in vivo by regulating FOXA1. Before animal experiments, SW480 cells were co-cultured with agomir-NC-exo and agomir-93-5p-exo, and nude mice were subcutaneously injected with these cells, and subjected to irradiation. **a** Quantitative analysis for tumor volume in nude mice. **b** Representative tumor images in nude mice. **c** Quantitative analysis for tumor weight in nude mice. **d**–**e** Positive expression of Bax and Bcl2 proteins determined by immunohistochemistry (400 ×). (F) miR-93-5p expression and FOXA1 mRNA expression in xenografted tumors determined by RT-qPCR, normalized to GAPDH. **g**–**h** FOXA1 protein bands and expression in xenografted tumors detected by Western blot analysis, normalized to GAPDH. Values obtained from three independent experiments in triplicate are expressed as mean ± SD and analyzed by one-way ANOVA followed by Tukey’s post hoc test among multiple groups, and repeated measures ANOVA followed by Bonferroni at different time points. *n* = 10. ^*^p < 0.05 nude mice injected with SW480 cells without treatment; ^#^*p* < 0.05 nude mice injected with SW480 cells transduced with agomir-NC-exo

In order to investigate whether the exosomes from CAFs transmit miR-93-5p to regulate FOXA1 gene and affect irradiation resistance in CRC, the nude mice were then subcutaneously implanted with cells that were cultured with serum-free medium containing agomir-NC-exo or agomir-93-5p-exo and subjected to irradiation. The volume and weight of tumors were increased in nude mice injected with SW480 cells transduced with agomir-NC-exo, and were significantly elevated in in nude mice injected with SW480 cells transduced with agomir-93-5p-exo (Fig. 7a–c).

Immunohistochemistry revealed that positive rate of Bax protein was reduced and that of Bcl2 protein was increased in nude mice injected with SW480 cells transduced with agomir-NC-exo, and the trends were more pronounced in nude mice injected with SW480 cells transduced with agomir-93-5p-exo (Fig. 7d–e).

RT-qPCR and Western blot analysis results revealed that miR-93-5p expression was elevated and FOXA1 expression was decreased in nude mice injected with SW480 cells transduced with agomir-NC-exo, and more variable changes were detected in nude mice injected with SW480 cells transduced with agomir-93-5p-exo (*p* < 0.05; Fig. 7f–h). These data provided evidence that overexpression of miR-93-5p in exosomes increased the tumor growth of CRC cells in vivo by inhibiting FOXA1.

## Discussion

It is known that the radioresistance of CRC cells leads to the failure of radiotherapy and toxic impacts of ionizing radiation [20]. Recent evidence has already demonstrated that miRNAs played an important role in radio-induced apoptosis and the radioresistance of CRC cells [21]. Exosomes were derived from various types of cells and contained parent cells-secreted miRNAs, which were involved in cancer therapies [22]. Therefore, our study investigated the role of CAFs-exo carrying miR-93-5p in radioresistance of CRC cells. Collectively, CAFs-exo carrying miR-93-5p functioned as a facilitator in radioresistance of CRC cells via their promotion in cell proliferation and colony formation and disruption in apoptosis of CRC cells through downregulating FOXA1 expression.

One important finding in our study was that miR-93-5p was highly expressed in CRC tissues and cells, while FOXA1 was poorly expressed. The aberrant expression of miRNAs is implicated in the development and progression of CRC [23]. Elevated miR-10b expression is found in CRC, which is correlated with the poor prognosis of patients with CRC [24]. miR-93 expression is reported to be increased in non-small lung cancer tissues [25]. A bioinformatics website in combination with a dual luciferase reporter gene assay validated that miR-93-5p cold target FOXA1, which was negatively regulated by miR-93-5p. FOXA1 could act as a tumor suppressor in human cancers, and its expression is associated with the prognosis of patients with cancers [17]. FOXL1 is also demonstrated to be downregulated in gallbladder cancer tissues and cells [26]. However, the decreased FOXL1 expression in CRC has not been reported. These evidences support that miR-93-5p was upregulated while FOXA1 was downregulated in CRC, and miR-93-5p displays a negative correlation with FOXA1.

In addition, we found that CRC cells could endocytose exosomes derived from CAFs which contained robust miR-93-5p. Exosomes have been previously described as secreted microvesicles that carry proteins, mRNAs and miRNAs by means of bodily fluids, which stimulate immune responses and accelerate communication among cells [27]. Ren et al. confirmed that CAFs could transfer exosomes to CRC cells, affecting tumor progression [7]. Meanwhile, various cell types-derived exosomal miRNAs were found to be correlated with metastatic niche preparation and tumor growth interference [28]. Exosomes derived from CAFs containing abundant miR-21 could be transferred into CRC cells [11], which supports our findings that CAFs show elevated miR-93-5p expression and deliver it to CRC cells through exosomes.

Furthermore, the data in the current study implied that exosome-mediated transfer of miR-93-5p from CAFs promoted CRC cell viability and colony formation and inhibited apoptosis to induce radioresistance in CRC cells by downregulating FOXA1 and upregulating TGFB3. It has been confirmed that elevated miR-106b could induces cell radioresistance in CRC [13]. CAFs transfer overexpressing miR-21 into CRC cells so as to rescue apoptosis and facilitate cell proliferation [29]. Consistent with this finding, exosome-mediated transfer of miR-21 enhances CRC cell proliferation and chemoresistance to promote the progression of CRC [11]. Moreover, the effects of FOXA1 in CRC cell proliferation and apoptosis have been revealed by an existing literature [17]. It has been proved that upregulation of FOXL1 inhibits cell proliferation in vitro and tumorigenicity in vivo and stimulates the apoptosis in gallbladder cancer [26]. In addition, FOXA1 is found to negatively regulate the transcription of TGFB3 in the current study. It has been indicated that knockdown of FOXA1 could upregulate TGFB3 to activate the TGF-β signaling pathway [19]. Those mentioned above are partially consistent with the most crucial finding of the current study, whereby CAFs-exo carrying miR-93-5p were identified to induce CRC cells resistant to radiotherapy by promoting cell proliferation and suppressing apoptosis in CRC by downregulating FOXA1 via activation of TGF-β signaling pathway.

## Conclusion

Taken together, CAFs-exo carrying miR-93-5p have the potential to serve as a promising target for miRNA-based therapy for CRC, due to its stimulatory effects on radioresistance in CRC cells by inducing tumor cell proliferation and colony formation and inhibiting cell apoptosis. Our investigation of CAFs-exo carrying miR-93-5p yielded promising results and an enhanced understanding regarding the molecular mechanism of carcinogenesis and progression of CRC. However, the research is still at the preclinical stage. In addition, the underlying role and mechanism of miR-93-5p in CRC remain to be elucidated. Thus, further investigations are needed to explore the relevant intrinsic mechanisms.

## Data Availability

All the data and materials are available.

## Abbreviations

ANOVA: analysis of variance
CAFs: cancer-associated fibroblasts
CCK-8: Cell counting kit-8
ChIP: Chromatin immunoprecipitation
CRC: Colorectal cancer
FBS: fetal bovine serum
FITC: V-fluorescein isothiocyanate
FOXA1: Forkhead box protein A1
GAPDH: glyceraldehyde-3-phosphate dehydrogenase
HIEC: Human normal intestinal epithelial cells
IgG: Immunoglobulin G
miRNAs: microRNAs
mRNAs: messenger RNAs
NAT: Nanoparticle tracking analysis
NFs: normal fibroblasts
OD: optical density
PBS: phosphate buffer saline
PI: propidium
RPMI: Roswell Park Memorial Institute
RT-qPCR: reverse transcription quantitative polymerase chain reaction
TEM: Transmission electron microscope
wt: wild type

## References

[1] Marmol I, Sanchez-de-Diego C, Pradilla Dieste A, Cerrada E, Rodriguez Yoldi MJ. Colorectal carcinoma: A general overview and future perspectives in colorectal cancer. Int J Mol Sci. 2017;18(1):E197. 10.3390/ijms18010197 PMC5297828 28106826

[2] Arnold M, Sierra MS, Laversanne M, Soerjomataram I, Jemal A, Bray F (2017). Global patterns and trends in colorectal cancer incidence and mortality. Gut..

[3] Yang P, Yang Y, An W, Xu J, Zhang G, Jie J (2017). The long noncoding rna-ror promotes the resistance of radiotherapy for human colorectal cancer cells by targeting the p53/mir-145 pathway. J Gastroenterol Hepatol.

[4] Wang D, Jiao C, Zhu Y, Liang D, Zao M, Meng X (2017). Activation of cxcl12/cxcr4 renders colorectal cancer cells less sensitive to radiotherapy via up-regulating the expression of survivin. Exp Biol Med (Maywood).

[5] Lotti F, Jarrar AM, Pai RK, Hitomi M, Lathia J, Mace A (2013). Chemotherapy activates cancer-associated fibroblasts to maintain colorectal cancer-initiating cells by il-17a. J Exp Med.

[6] Gonda TA, Varro A, Wang TC, Tycko B (2010). Molecular biology of cancer-associated fibroblasts: can these cells be targeted in anti-cancer therapy?. Semin Cell Dev Biol.

[7] Ren J, Ding L, Zhang D, Shi G, Xu Q, Shen S (2018). Carcinoma-associated fibroblasts promote the stemness and chemoresistance of colorectal cancer by transferring exosomal lncrna h19. Theranostics..

[8] Arscott WT, Tandle AT, Zhao S, Shabason JE, Gordon IK, Schlaff CD (2013). Ionizing radiation and glioblastoma exosomes: implications in tumor biology and cell migration. Transl Oncol.

[9] Ono M, Kosaka N, Tominaga N, Yoshioka Y, Takeshita F, Takahashi RU (2014). Exosomes from bone marrow mesenchymal stem cells contain a microrna that promotes dormancy in metastatic breast cancer cells. Sci Signal..

[10] Lasser C (2012). Exosomal rna as biomarkers and the therapeutic potential of exosome vectors. Expert Opin Biol Ther.

[11] Bhome R, Goh RW, Bullock MD, Pillar N, Thirdborough SM, Mellone M (2017). Exosomal micrornas derived from colorectal cancer-associated fibroblasts: role in driving cancer progression. Aging (Albany NY).

[12] Sun C, Wang FJ, Zhang HG, Xu XZ, Jia RC, Yao L (2017). Mir-34a mediates oxaliplatin resistance of colorectal cancer cells by inhibiting macroautophagy via transforming growth factor-beta/smad4 pathway. World J Gastroenterol.

[13] Zheng L, Zhang Y, Liu Y, Zhou M, Lu Y, Yuan L (2015). Mir-106b induces cell radioresistance via the pten/pi3k/akt pathways and p21 in colorectal cancer. J Transl Med.

[14] Yang X, Xu X, Zhu J, Zhang S, Wu Y, Wu Y (2016). Mir-31 affects colorectal cancer cells by inhibiting autophagy in cancer-associated fibroblasts. Oncotarget..

[15] Xiang Y, Liao XH, Yu CX, Yao A, Qin H, Li JP (2017). Mir-93-5p inhibits the emt of breast cancer cells via targeting mkl-1 and stat3. Exp Cell Res.

[16] Bernardo GM, Keri RA (2012). Foxa1: a transcription factor with parallel functions in development and cancer. Biosci Rep.

[17] Ma W, Jiang J, Li M, Wang H, Zhang H, He X (2016). The clinical significance of forkhead box protein a1 and its role in colorectal cancer. Mol Med Rep.

[18] Hisamatsu Y, Tokunaga E, Yamashita N, Akiyoshi S, Okada S, Nakashima Y (2012). Impact of foxa1 expression on the prognosis of patients with hormone receptor-positive breast cancer. Ann Surg Oncol.

[19] Song B, Park SH, Zhao JC, Fong KW, Li S, Lee Y (2019). Targeting foxa1-mediated repression of tgf-beta signaling suppresses castration-resistant prostate cancer progression. J Clin Invest.

[20] Khoshinani HM, Afshar S, Pashaki AS, Mahdavinezhad A, Nikzad S, Najafi R (2017). Involvement of mir-155/foxo3a and mir-222/pten in acquired radioresistance of colorectal cancer cell line. Jpn J Radiol.

[21] Zou Y, Yao S, Chen X, Liu D, Wang J, Yuan X (2018). Lncrna oip5-as1 regulates radioresistance by targeting dyrk1a through mir-369-3p in colorectal cancer cells. Eur J Cell Biol.

[22] Xu YF, Hannafon BN, Zhao YD, Postier RG, Ding WQ (2017). Plasma exosome mir-196a and mir-1246 are potential indicators of localized pancreatic cancer. Oncotarget..

[23] Corte H, Manceau G, Blons H, Laurent-Puig P (2012). Microrna and colorectal cancer. Dig Liver Dis.

[24] Nishida N, Yamashita S, Mimori K, Sudo T, Tanaka F, Shibata K (2012). Microrna-10b is a prognostic indicator in colorectal cancer and confers resistance to the chemotherapeutic agent 5-fluorouracil in colorectal cancer cells. Ann Surg Oncol.

[25] Zhu W, He J, Chen D, Zhang B, Xu L, Ma H (2014). Expression of mir-29c, mir-93, and mir-429 as potential biomarkers for detection of early stage non-small lung cancer. PLoS One.

[26] Qin Y, Gong W, Zhang M, Wang J, Tang Z, Quan Z (2014). Forkhead box l1 is frequently downregulated in gallbladder cancer and inhibits cell growth through apoptosis induction by mitochondrial dysfunction. PLoS One.

[27] McDonald MK, Tian Y, Qureshi RA, Gormley M, Ertel A, Gao R (2014). Functional significance of macrophage-derived exosomes in inflammation and pain. Pain..

[28] Sempere LF, Keto J, Fabbri M (2017). Exosomal micrornas in breast cancer towards diagnostic and therapeutic applications. Cancers (Basel).

[29] Bullock MD, Pickard KM, Nielsen BS, Sayan AE, Jenei V, Mellone M (2013). Pleiotropic actions of mir-21 highlight the critical role of deregulated stromal micrornas during colorectal cancer progression. Cell Death Dis.

